# Quantitative analysis of low-density SNP data for parentage assignment and estimation of family contributions to pooled samples

**DOI:** 10.1186/s12711-014-0051-y

**Published:** 2014-09-02

**Authors:** John M Henshall, Leanne Dierens, Melony J Sellars

**Affiliations:** CSIRO Food Futures National Research Flagship, CSIRO Animal, Food and Health Sciences, Armidale, NSW 2350 Australia; CSIRO Food Futures National Research Flagship, CSIRO Animal, Food and Health Sciences, St Lucia Qld, 4067 Australia; CSIRO Food Futures National Research Flagship, CSIRO Marine and Atmospheric Research, Dutton Park, Qld, 4102 Australia

## Abstract

**Background:**

While much attention has focused on the development of high-density single nucleotide polymorphism (SNP) assays, the costs of developing and running low-density assays have fallen dramatically. This makes it feasible to develop and apply SNP assays for agricultural species beyond the major livestock species. Although low-cost low-density assays may not have the accuracy of the high-density assays widely used in human and livestock species, we show that when combined with statistical analysis approaches that use quantitative instead of discrete genotypes, their utility may be improved. The data used in this study are from a 63-SNP marker Sequenom® iPLEX Platinum panel for the Black Tiger shrimp, for which high-density SNP assays are not currently available.

**Results:**

For quantitative genotypes that could be estimated, in 5% of cases the most likely genotype for an individual at a SNP had a probability of less than 0.99. Matrix formulations of maximum likelihood equations for parentage assignment were developed for the quantitative genotypes and also for discrete genotypes perturbed by an assumed error term. Assignment rates that were based on maximum likelihood with quantitative genotypes were similar to those based on maximum likelihood with perturbed genotypes but, for more than 50% of cases, the two methods resulted in individuals being assigned to different families. Treating genotypes as quantitative values allows the same analysis framework to be used for pooled samples of DNA from multiple individuals. Resulting correlations between allele frequency estimates from pooled DNA and individual samples were consistently greater than 0.90, and as high as 0.97 for some pools. Estimates of family contributions to the pools based on quantitative genotypes in pooled DNA had a correlation of 0.85 with estimates of contributions from DNA-derived pedigree.

**Conclusions:**

Even with low numbers of SNPs of variable quality, parentage testing and family assignment from pooled samples are sufficiently accurate to provide useful information for a breeding program. Treating genotypes as quantitative values is an alternative to perturbing genotypes using an assumed error distribution, but can produce very different results. An understanding of the distribution of the error is required for SNP genotyping platforms.

**Electronic supplementary material:**

The online version of this article (doi:10.1186/s12711-014-0051-y) contains supplementary material, which is available to authorized users.

## Background

High-density SNP (single nucleotide polymorphism) assays have become a standard tool in the study of human, animal and plant genetics, due to a rapid increase in SNP density for a constant or falling price per assay. Concurrently, progress has been achieved in technologies for low-density SNP assays with a substantially reduced price per assay. These developments in two directions i.e. maximum density per unit of cost and minimum cost per assay, have provided tools at different price points that are suited to different applications across a broad range of genetics studies. While much of the focus in the literature is on discoveries that arise from the application of the newest, most powerful high-density assays, in many applications, especially in species other than humans, reducing the price per assay is more important than increasing the number of SNPs in the assay. Parentage assignment in commercial livestock industries is an example. The benefit of knowing pedigree is real and quantifiable, but is relatively small per individual animal, and the cost of alternative means of assigning parentage puts a cap on the price at which genomic assays will be beneficial. In commercial aquaculture there are often no alternative means of assigning parentage unless families are reared in isolation, which is undesirable from the perspective of tank infrastructure. Consequently, there is much interest in the use of genomic assays for pedigree construction. In aquaculture, the value of individual animals is perceived to be lower than in many terrestrial livestock species, so systems to assign parentage may be even more sensitive to the price of assays. However, the opportunities are significant. DNA-based parentage removes the need for single-family rearing, allows physical tagging at a much later age, and offers the opportunity to link phenotypes from individuals grown in commercial ponds to broodstock grown in specialized pond or tank systems [1]. In cases where recording pedigree through management from mating to tagging is impractical, the introduction of molecular-based parentage offers perhaps the only opportunity to embark on a modern genetic improvement program. Importantly, this does not necessarily require the use of high-density genotyping arrays.

The shrimp industry is an example of an industry for which to date no high-density SNP assays have been developed, despite ongoing efforts. Establishment of Penaeid shrimp selective breeding programs over the last 20 years have underpinned global expansion of a sustainable shrimp aquaculture industry that is worth an estimated US 3 billion dollars in 2012 (MRP Briggs, personal communication). More recently, breeding programs have adopted microsatellite-based DNA testing to enable accurate pedigree assignment, both to select for specific genetic traits that maximise production efficiency and to restrict inbreeding [2–5]. When applying molecular tools to parentage testing, statistical methods developed in other disciplines have usually been used. For example, in the analysis of SNP data from a designed Black Tiger shrimp *Penaeus monodon* breeding program, Sellars *et al.* [5] applied maximum likelihood methods that were developed for parentage assignment using microsatellites in wild populations. Both the change from microsatellites to SNPs and differences between wild populations and managed breeding populations provide justification for reviewing the appropriateness of these existing methods for parentage assignment.

Microsatellites have been empirically compared to SNPs for parentage analysis in a number of studies e.g. [5–8] and parentage assignment using SNPs is discussed in other studies, e.g. [9–12]. Earlier studies favoured microsatellites, especially for non-model organisms, due to the ease of developing microsatellites and the additional power that results from the larger numbers of alleles at each locus. However, the price per SNP in a high-density array is so much less than the price of an equivalent amount of information from microsatellites that more recent studies accept that SNPs can be more efficient in some circumstances. If SNPs are sufficiently closely linked, haplotypes of SNPs that are in linkage disequilibrium can be estimated, and these are far more polymorphic than individual SNPs and can be treated as alleles in parentage analyses [11,13]. In these situations, the differences in polymorphism between SNPs and microsatellites become negligible.

To date, comparisons of microsatellites and SNPs for parentage analysis have generally used the same methods and software for each assay platform, with little consideration of the properties of the method. Over the years, a number of statistical approaches to parentage analysis have been considered (see Jones *et al.* [9] for a review). The aim of the current study is the quantitative analysis of genotypes from low-cost, low-density SNP assays, focusing on maximum likelihood based methods that allow for the possibility of genotyping errors, e.g. [14–20]. The likely form of genotyping errors in bi-allelic SNP data was explicitly discussed by Sieberts *et al.* [15], Anderson and Garza [10] and Teo *et al.* [21]. With only two alleles at each locus, a genotyping error is easily modelled as the probability of an allele being read as the alternative allele, and this probability can be assumed to be constant across SNPs and samples, or specific to SNPs or samples, e.g. [21]. A second reason for focusing on maximum likelihood methods is that the likelihoods of alternative pedigrees can serve as input into a second analysis, in which other, non genetic data is included [22,23]. For example, the probability of a particular mating having occurred can be affected by spatial location records, both in wild and in managed populations.

In all studies mentioned above, assay results from microsatellites or SNPs were modelled as discrete pairs of alleles. However, in their raw form, SNP assays return quantitative estimates of area or intensity for two axes, X and Y, which relate to the two nucleotide alleles. For example, the Illumina BEADSTUDIO software can be configured to return the frequency of the B allele, a quantitative measure taking values between 0 and 1 [24]. Commonly, these quantitative values are used to estimate SNP allele frequencies in pooled samples of DNA, which can then be used to estimate SNP effects on disease or production traits, e.g. [1,25–30], but they can also be used to estimate population parameters, such as the proportional contributions from multiple families in a pooled sample [31]. This application is of particular interest for selective breeding in aquaculture, where individual animals are of low value, but family numbers are very large. In shrimp, genotyping assays of pooled DNA samples provide promise for linking substantial amounts of quantitative phenotypic data from commercial ponds to parental broodstock. High-performing families can be identified and young broodstock from these families can then be used to produce the next generation.

In this study, we attempted to incorporate all of the seemingly disparate aspects of SNPs and parentage analysis noted above into a single approach. We used a low-density SNP panel to genotype individuals and pools from several generations from a domesticated *P. monodon* breeding population. Instead of applying an error function to discrete allele calls, we estimated genotype probabilities directly from the quantitative X-Y data from the assay, using SNP specific parameters that were calibrated from as many samples as are available. These genotype probabilities were then used in maximum likelihood parentage analysis, using a matrix formulation to simplify the algebra and reduce the risk of software errors. We also describe the matrix formulation of a traditional maximum likelihood method [19] and of simply counting allele mismatches and, for comparison, applied them to the same dataset. The likelihoods from the parentage analysis were used to identify likely family structures that are consistent with known biological constraints in *P. monodon*. Finally, we used the most likely parent pairs identified in this analysis to estimate family contributions in pooled samples of DNA from the progeny.

## Methods

A worked example of the statistical approach used in this study is in the Appendix.

### Animals, tissue sampling, DNA extraction and SNP genotyping

Samples were available from multiple generations of *P. monodon* shrimp from a single hatchery. All available pleopod tips (approximately 8 mm^2^ in size) from G9 (generation 9, n = 311) breeding shrimp and a random sample of 202 G10 shrimp from the same shrimp breeding line were used. Pleopod tips were snap-frozen on dry ice for transport back to the laboratory. Genomic DNA (gDNA) was extracted from each individual using CSIRO standard industry protocols that apply QIAGEN’s DNeasy extraction methodology. gDNA was also extracted from 583 G1 and G2 individuals from an unrelated breeding line from the same farm. In addition to the individual samples, the 202 G10 shrimp were allocated at random to nine pools, each containing between 18 and 24 individuals, and the 311 G9 shrimp were allocated at random to 13 pools, each containing between 23 and 26 individuals. Pooled gDNA samples were then created by mixing 5 μL of gDNA from each individual in the pool. Subsequently, 60 μL from each pooled samples and 500 ng of gDNA from each individual shrimp were sent to GeneWorks® for genotyping using the 63-SNP marker Sequenom® iPLEX Platinum panel reported by Sellars *et al.*, [32]. Of these 63 SNPs, two consistently failed and were removed from all analyses, leaving a 61-SNP assay.

### Estimation of SNP specific parameters

All 1096 G1, G2, G9 and G10 individual samples (i.e., excluding the samples of pooled DNA) were used to estimate SNP specific parameters. In addition to the standard genotype call for each SNP for each sample, GeneWorks® provided estimates of the X and Y areas that relate to the A and B alleles for each SNP, which are the raw output from the Sequenom genotyping platform. For assay *ij*, for sample *i* and SNP *j*, these consist of an area for each allele (*a*_*1ij*_ and *a*_*2ij*_) and an uncertainty measure for each allele (*u*_*1ij*_ and *u*_*2ij*_). We excluded assay results when the Sequenom software failed to call an allele. For all other assays, we converted Cartesian X-Y area measurements to polar coordinates [24] to derive a measure of allelic proportion as:

1 $$ {p}_{ij} = \frac{{ \tan}^{-1}\left(\frac{a_{2 ij}-{u}_{2 ij}}{a_{1 ij}-{u}_{1 ij}}\right)}{\frac{\pi }{2}}. $$

This can be interpreted as a measure taking a value between 0.0 and 1.0, corresponding to the polar coordinate range from 0 to $\frac{\pi }{2}$. A figure showing the relationship between the X and Y areas and *p*_*ij*_ is in the Appendix. We then used the genotype calls from the Sequenom software to estimate means (*μ*_AA*j*_, *μ*_AB*j*_ and *μ*_BB*j*_) and standard deviations (*σ*_AA*j*_, *σ*_AB*j*_ and *σ*_BB*j*_) for values *p*_*ij*_ for each genotype class (homozygous AA, heterozygous AB, and homozygous BB) for each SNP. Unlike in the approach used to estimate B allele frequencies in [24], where allelic proportions were scaled such that values *p*_*ij*_ = 0.0, 0.5 and 1.0 corresponded to the means *μ*_AA*j*_, *μ*_AB*j*_ and *μ*_BB*j*_, respectively, we did not scale the *p*_*ij*_ values. As a measure of the power to discriminate between genotype classes, we used the Welch statistic (the t-statistic appropriate when the variances of classes are unequal, using the standard output for the t.test() function in R [33]). The Welch statistics τ_A*j*_ and τ_B*j*_ were estimated for the intervals (*μ*_AA*j*_ to *μ*_AB*j*_) and (*μ*_AB*j*_ to *μ*_BB*j*_), respectively.

### Parentage assignment

Parents from generation G9 were assigned to the G10 individuals in a two-step process.

First, the likelihoods of sire-dam-offspring trios were estimated. Instead of first assigning genotypes and then applying a likelihood method that incorporated an error term (such as in [19,34,35]), the likelihood method explicitly incorporated the SNP specific parameters (*μ*_AA*j*_, *μ*_AB*j*_, *μ*_BB*j*_,*σ*_AA*j*_, *σ*_AB*j*_ and *σ*_BB*j*_) and the measures of allelic proportion *p*_*ij*_. The latter were included regardless of whether the Sequenom software called a genotype, but were set to missing when *a*_*1ij*_ - *u*_*1ij*_ + *a*_*2ij*_ - *u*_*2ij*_ was less than 3.0. This cut-off was chosen after examining the values of *a* and *u* for those samples that were not assigned a genotype by the Sequenom software. For each *p*_*ij*_, a quantitative ordered genotype probability matrix **G**^**ij**^ was estimated as:

$$ {\mathbf{G}}^{\mathbf{ij}}=\left[\begin{array}{cc}\hfill {\Phi}_{\mathrm{AA} ij}\hfill & \hfill {\Phi}_{\mathrm{AB} ij}\hfill \\ {}\hfill {\Phi}_{\mathrm{BA} ij}\hfill & \hfill {\Phi}_{\mathrm{BB} ij}\hfill \end{array}\right]/{\displaystyle \sum }{\Phi}_{ij}, $$

where Φ_AA*ij*_ is the height of the N(*μ*_AA*j*_, σ_AA*j*_) distribution at *x* = *p*_*ij*_, Φ_AB*ij*_ and Φ_BA*ij*_ are each half the height of the N(*μ*_AB*j*_, *σ*_AB*j*_) distribution at *x* = *p*_*ij*_, Φ_BB*ij*_ is the height of the N(*μ*_BB*j*_, *σ*_BB*j*_) distribution at *x* = *p*_*ij*_, and the sum ΣΦ_*ij*_ is over genotypes AA, AB, BA and BB. The **G** matrices are symmetric, and have dimension (2 × 2) for biallelic SNPs. The vector of probabilities of allele transmission from a sire or dam *i* at marker *j* is

2 $$ {\mathbf{T}}^{\mathbf{i}\mathbf{j}}=\left({\mathbf{G}}^{\mathbf{i}\mathbf{j}}\left[\begin{array}{c}\hfill 1\hfill \\ {}\hfill 1\hfill \end{array}\right]+{\mathbf{G}}^{\mathbf{i}{\mathbf{j}}^{\hbox{'}}}\ \left[\begin{array}{c}\hfill 1\hfill \\ {}\hfill 1\hfill \end{array}\right]\right)/2. $$

Multiplying by the vector of 1s produces the row averages $$ \left({\mathbf{G}}^{\mathbf{ij}}\left[\begin{array}{c}\hfill 1\hfill \\ {}\hfill 1\hfill \end{array}\right]\right) $$ and the column averages $$ \left({\mathbf{G}}^{\mathbf{i}{\mathbf{j}}^{\hbox{'}}}\ \left[\begin{array}{c}\hfill 1\hfill \\ {}\hfill 1\hfill \end{array}\right]\right) $$, which are equal because **G** is symmetric. So here the equation for **T** could be simplified, but there are situations where **G** might not be symmetric (for example, in a multigenerational dataset where parental origin of alleles is known), so we chose to leave the equation in this more general format. For a potential parent pair, sire (s) and dam (d), with transmission vectors **T**^**sj**^ and **T**^**dj**^, the transmission matrix ${\mathbf{T}}^{\mathbf{sj}}{\mathbf{T}}^{\mathbf{d}{\mathbf{j}}^{\hbox{'}}}$ contains the genotype probabilities expected from that pairing. Thus, for offspring (o) with genotype probability matrix **G**^**oj**^, the likelihood of the trio (*s, d, o*) for marker *j* is:

3 $$ {L}^{(sdo)j}=\mathrm{sum}\left(\left({\mathbf{T}}^{\mathbf{sj}}{\mathbf{T}}^{\mathbf{d}{\mathbf{j}}^{\hbox{'}}}\right){}^{\circ}{\mathbf{G}}^{\mathbf{oj}}\right), $$

where *°* is the Hadamard or entrywise product. To estimate the likelihood when marker data for one parent is missing, the genotype matrix for the missing parent was set to ${\mathbf{F}}^{\mathbf{j}}{\mathbf{F}}^{{\mathbf{j}}^{\hbox{'}}}$, where **F**^**j**^ is the (2 × 1) vector of allele frequencies for SNP *j*, estimated from all G9 animals. In the likelihood ratio $\frac{L^{(sdo)j}}{L^{oj}}$, the denominator *L*^*oj*^ is the likelihood under the null hypothesis that the offspring is unrelated to the sire and dam, which is constructed by treating both parents as missing. The likelihood ratio across all markers is the product of the marker likelihood ratios, or the sum of the log-likelihood ratios. For consistency with earlier publications, we will refer to the summed log-likelihood as the log odds (LOD) score. We only retained LOD scores where, for at least 10 SNPs, none of the sire, dam or offspring genotypes were missing. This relatively relaxed threshold was used because the LOD scores were further processed in the second step, as described in a following paragraph. When the LOD for a sire-dam-offspring trio was less than the LOD for the sire-offspring pair with a missing dam, the sire-offspring LOD was used and the dam was assumed to be missing. Likewise, when the LOD for a sire-dam-offspring trio was less than the LOD for the dam-offspring pair with a missing sire, the dam-offspring LOD was used and the sire was assumed to be missing. The most likely pedigree was identified and will be referred to as the unrestricted pedigree.

The second step in the parentage assignment was to impose constraints due to the reproductive biology of *P. monodon* and hatchery records. The G10 offspring were produced by natural mating of the G9 parents in large mating groups, spawned over a three-week period. Under natural mating, females can only mate immediately following moult, which occurs in a cycle of approximately three weeks, and at each moult, females mate with at most one male. Given the width of the spawning window, it is unlikely, but not impossible, that females produced offspring from more than one male. After mating, male *P. monodon* take 7 to 12 days to re-develop their spermatophores [36,37]. So similarly, it is unlikely, but not impossible, that males produced offspring from more than one female. Accordingly, we used a stochastic search process to identify two constrained pedigrees, first the set of full-sib families that maximised the total LOD (referred to as the full-sib pedigree) and second, the set of half-sib families that maximised the total LOD (referred to as the half-sib pedigree), where for the half-sib families, parents could have no more than two mates. For each full-sib and half-sib pedigree, 5000 independent random pedigrees were sampled and the one with the maximum total LOD retained. Each random pedigree was produced by proceeding through randomly ordered offspring, creating a family for the sire-dam-offspring mating pair with the highest LOD that did not violate mating constraints. With this approach, the largest families are likely to be identified first, maximising the chance of finding a high likelihood solution for the whole pedigree. We validated the method by repeating the process five times, and in each case the same solution was found, always within the first 1000 of the 5000 samples. To assist in determining whether departures from the hypothesized full-sib pedigree structure were due to lack of power in parentage assignment or to parents having multiple mates, we fitted two generalised linear models, with the response vector for the first model coded 0/1 when the parent pair was different/the same in the unrestricted and half-sib pedigrees, and with the response vector for the second model coded 0/1 when the parent pair was different/the same in the half-sib and full-sib pedigrees. For each offspring, the explanatory variable was the sum of the genotype probabilities for the most likely unordered genotype for each SNP.

### Matrix formulation of maximum likelihood and exclusion methods

For comparison, we also conducted the analysis described above using an established maximum likelihood approach [19,34,35] and by exclusion. Both of these are easily represented in matrix form. For the maximum likelihood approach, the genotype probability matrix is built as ${\mathbf{M}}^{\mathbf{ij}} = \left(1-{\varepsilon}_j\right){\mathbf{D}}^{\mathbf{ij}}+{\varepsilon}_j{\mathbf{F}}^{\mathbf{j}}{\mathbf{F}}^{{\mathbf{j}}^{\hbox{'}}}$, where the discrete genotype matrix $$ {\mathbf{D}}^{\mathbf{ij}}=\left[\begin{array}{cc}\hfill 1\hfill & \hfill 0\hfill \\ {}\hfill 0\hfill & \hfill 0\hfill \end{array}\right],\left[\begin{array}{cc}\hfill 0\hfill & \hfill 1\hfill \\ {}\hfill 0\hfill & \hfill 0\hfill \end{array}\right]\ \mathrm{or}\ \left[\begin{array}{cc}\hfill 0\hfill & \hfill 0\hfill \\ {}\hfill 0\hfill & \hfill 1\hfill \end{array}\right] $$, the genotype probability matrix expected due to the allele frequency is ${\mathbf{F}}^{\mathbf{j}}{\mathbf{F}}^{{\mathbf{j}}^{\hbox{'}}}$, as described above, and ε is the assumed error rate. Alternative formulations of the matrix for heterozygous individuals are equivalent, provided that the sum of the heterozygous probabilities equals 1, while the assumed error rates may be constant or differ between SNPs. We estimated SNP specific error rates in two different ways. First, as $\widehat{\varepsilon_J}= mean\left(1-{g}_{.j}^{max}\right)$, where ${g}_{.j}^{max}$ is the vector for SNP *j* that comprised the maximums of the unordered genotypes in **G**^**ij**^ (with the unordered heterozygous genotype being the sum of the two ordered heterozygous genotypes); and second, as:

$$ {\tilde{\varepsilon}}_J = \left\{\begin{array}{c}\hfill \widehat{\varepsilon_J},\kern2.75em \widehat{\varepsilon_J}>0.01\ \hfill \\ {}\hfill 0.01,\kern1.25em \widehat{\varepsilon_J}\le 0.01\hfill \end{array}\right.. $$

This second formulation allows for an underlying error rate regardless of the precision of the genotyping assay, which is similar to allowing a certain number of mismatches when using an exclusion method. We refer to $\widehat{\varepsilon}$ and $\tilde{\varepsilon}$ as the estimated and assumed error rates, respectively.

To assign parentage, the genotype probability matrix **M**^**ij**^ replaces the genotype probability matrix **G**^**ij**^ in Equation 2, while no changes to Equation 3 are required. We refer to this maximum likelihood method as “perturbed”, since the **D**^**ij**^ matrices are perturbed with an assumed error function. In forming the **D**^**ij**^ matrices, we included only markers for which the most likely genotype in the quantitative ordered genotype probability matrix **G**^**ij**^ was greater than 0.98 (or 0.49 for the two heterozygous genotypes).

Counting mismatches for the exclusion method using the matrix formulation is equally straightforward. Matrix **D**^**ij**^ is used instead of **M**^**ij**^ to produce the transition matrices in Equation 2 and, similar to the likelihood calculation in Equation 3, the number of mismatches is the number of markers for which $sum\left(\left({\mathbf{T}}^{\mathbf{sj}}{\mathbf{T}}^{\mathbf{d}{\mathbf{j}}^{\hbox{'}}}\right){}^{\circ}{\mathbf{D}}^{\mathbf{oj}}\right)$ equals 0. In assigning parents, we either allowed for no or three mismatches. Three mismatches out of 61 SNPs is small relative to the 15 out of 122 SNPs allowed by [6], but SNPs have only two alleles, so mismatches between unrelated individuals are expected to occur by chance far less frequently with SNPs than with microsatellites.

### Estimation of allele frequencies for pooled samples

For each SNP (subscripted *j*), for each of the nine pools (subscript *k*) we assumed that the reaction had failed for assays for which *a*_1*kj*_ + *a*_2*kj*_ − *u*_1*kj*_ − *u*_2*kj*_ ≤ 3. For the remaining assays, allelic proportions *p*_*kj*_ were estimated for each pooled sample using Equation 1. As in Peiffer *et al*. [24], the genotype means (*μ*_AA*j*_, *μ*_AB*j*_ and *μ*_BB*j*_) estimated from individual samples were used to adjust the allelic proportions to obtain estimates of allele frequencies $\widehat{f_{kJ}}$, where

$$ \widehat{f_{kJ}}=\left\{\begin{array}{ll}0.0\hfill & \mathrm{if}\ {\mathrm{p}}_{\mathrm{kj}}<{\mu}_{\mathrm{AA}j}\hfill \\ {}0.5\left(\frac{\left({p}_{kj}-{\mu}_{\mathrm{AA}j}\right)}{\left({\mu}_{\mathrm{AB}j}-{\mu}_{\mathrm{AA}j}\right)}\right)\hfill & \mathrm{if}\ {\mu}_{\mathrm{AA}j} < {p}_{kj}<{\mu}_{\mathrm{AB}j}\hfill \\ {}0.5+0.5\left(\frac{\left({p}_{kj}-{\mu}_{\mathrm{AB}j}\right)}{\left({\mu}_{\mathrm{BB}j}-{\mu}_{\mathrm{AB}j}\right)}\right)\hfill & \mathrm{if}\ {\mu}_{\mathrm{AB}j} < {p}_{kj}<{\mu}_{\mathrm{BB}j}\hfill \\ {}1.0\hfill & \mathrm{if}\ {p}_{kj}>{\mu}_{\mathrm{BB}j}\hfill \end{array}\right.. $$

As an estimate of the variation associated with $\widehat{f_{kJ}}$, we used the relevant Welch statistic (τ_A*j*_ or τ_B*j*_):

$$ {\tau}_{kj} = \left\{\begin{array}{c}\hfill {\uptau}_{Aj}\kern1.5em \mathrm{if}\ {p}_{kj}\le {\mu}_{\mathrm{AB}j}\ \hfill \\ {}\hfill {\uptau}_{Bj}\kern1.5em \mathrm{if}\;{p}_{kj}>{\mu}_{\mathrm{AB}j}\hfill \end{array}\right.. $$

Allele frequencies of pooled samples, ${f}_{kj}^{*}$, were also estimated from the genotype probability matrices of the individuals that were used to create each pool. This was done using the estimated allelic proportions for the individuals. The correlation between $\widehat{f_{kj}}$ and ${f}_{kj}^{*}$ was estimated for each pool, and the effect of genotyping success rates on the correlation was tested using a regression analysis.

### Estimation of family contributions to pools

The families represented in the nine G10 pools were determined using the known G10 individuals in the pool and the full-sib, half-sib and unrestricted pedigrees were identified using the stochastic search described above. With *m* being the number of SNPs for each pool *k*, containing individuals from *n* families, matrices **X** (subscript *k* implied), **y**, and **w** were assembled as follows:

$$ \mathbf{X}=\left[\begin{array}{cccc}\hfill {X}_{11}\hfill & \hfill {X}_{12}\hfill & \hfill \cdots \hfill & \hfill {X}_{1n}\hfill \\ {}\hfill {X}_{21}\hfill & \hfill {X}_{22}\hfill & \hfill \cdots \hfill & \hfill {X}_{2n}\hfill \\ {}\hfill \vdots \hfill & \hfill \vdots \hfill & \hfill\ \hfill & \hfill \vdots \hfill \\ {}\hfill {X}_{m1}\hfill & \hfill {X}_{m2}\hfill & \hfill \cdots \hfill & \hfill {X}_{mn}\hfill \end{array}\right], $$

$$ \mathbf{y}=\left[\begin{array}{c}\hfill \widehat{f_{k1}}\hfill \\ {}\hfill \widehat{f_{k2}}\hfill \\ {}\hfill \vdots \hfill \\ {}\hfill \widehat{f_{km}}\hfill \end{array}\right], $$

$$ \mathbf{w}=\left[\begin{array}{c}\hfill {\left({\tau}_{k1}^{est}\right)}^2\hfill \\ {}\hfill {\left({\tau}_{k2}^{est}\right)}^2\hfill \\ {}\hfill \vdots \hfill \\ {}\hfill {\left({\tau}_{km}^{est}\right)}^2\hfill \end{array}\right], $$

where *X*_*jl*_ contains estimates of the allele frequencies for SNP *j* and family *l*, given the estimated genotype probability matrices for the sire and dam described above. Missing values in **X** and **y** were replaced with 0.5, and missing values in **w** were replaced with 1.0. These **X** and **y** matrices are equivalent to those of Kinghorn *et al.* [31], except that we add a vector of weights **w**. These weights are based on the differences in resolution between genotype clusters for individual samples, which we assume is related to the variance of allele frequency estimates for pooled samples. We then used the pcls() function in the R package mgcv [33,38] to solve the weighted least squares problem $\mathit{\min}{\left\Vert \sqrt{\mathbf{w}}\left(\mathbf{X}\boldsymbol{\beta } -\mathbf{y}\right)\right\Vert}^2$ for the (*n* × 1) vector of family contributions ***β***, subject to the constraint *min*(***β***) > 0. Strictly, an additional constraint ∑ ***β*** = 1.0 applies, but we found that the sum of the solution vector β* returned by pcls() was generally close to 1.0 (min = 1.02, max = 1.12), and we scaled β*  to produce a final estimated family contribution vector $\widehat{\boldsymbol{\beta}} = {\boldsymbol{\beta}}^{*}/{\displaystyle \sum }{\boldsymbol{\beta}}^{*}$. For each pool, we also constructed vectors **y** using the means of allele frequencies for individual samples in the pool (i.e., using ${f}_{kj}^{*}$ instead of $\widehat{f_{kJ}}$) and we estimated family contributions from these for comparison.

## Results

### SNP genotyping and estimation of SNP specific parameters

**Figure 1:**
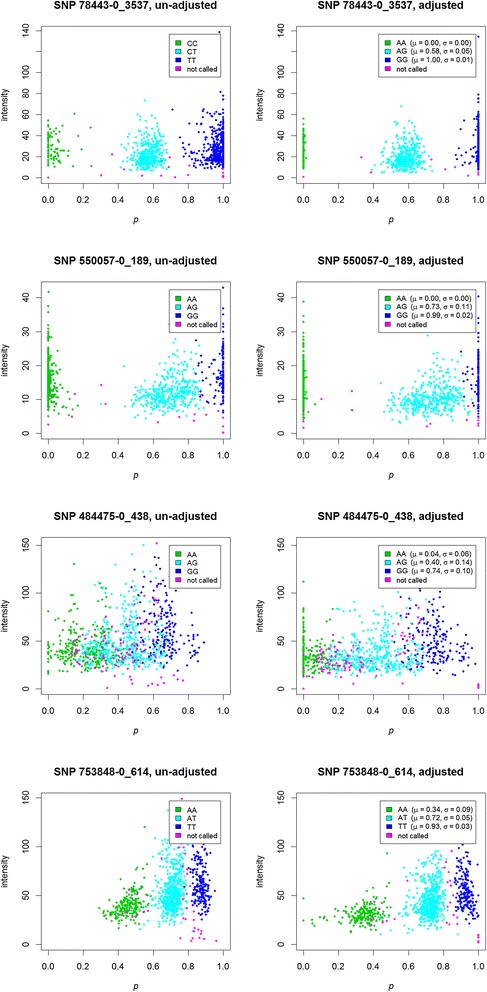
**Variation between SNPs and effect of adjusting for uncertainty.** Intensity is plotted against allelic proportion *p* (see Equation 1) for all individuals for four selected SNPs, using both the unadjusted areas (left hand side) and areas adjusted for the uncertainty associated with the area estimate as provided by the genotyping provider (right hand side); intensities are estimated as Euclidean distances from the origin to the data points in Cartesian coordinates; for the adjusted areas, the mean and standard deviation of the allelic proportion estimates (*p*) are provided.

Of the 64 538 assays (1058 G1, G2, G9, and G10 animals by 61 SNPs), an estimated genotype was returned by the Sequenom software for 54 761 assays. There was a lot of variation in genotype call rates between SNPs. The reasons for this become evident when the raw data are examined. In Figure 1, intensity is plotted against allelic proportion (*p*) for selected SNPs, based on X-Y areas unadjusted for area uncertainty on the left, and based on X-Y areas adjusted for area uncertainty on the right. For the adjusted plots, the means (*μ*_AA*j*_, *μ*_AB*j*_ and *μ*_BB*j*_) and standard deviations (*σ*_AA*j*_, *σ*_AB*j*_ and *σ*_BB*j*_) from values *p*_*ij*_ in each genotype class (AA, AB, and BB) are also provided. The first SNP (78443-0_3537) illustrates the value in subtracting the uncertainty value; although there was little change in the values of *p* for heterozygous individuals, the variation in the values of *p* for homozygous individuals was much reduced. The second SNP (550057-0_189) illustrates that the variation in heterozygous individuals could be much higher than that in homozygous individuals, and that the mean *p* for heterozygous individuals may not lie half way between the means for the homozygous individuals. For this SNP, most assays produced a genotype estimate. The third SNP (484475-0_438) exhibits even more variation, and the mean value of *p* for homozygous GG individuals was not close to 1.0. For this SNP, a genotype was not returned for some assays when *p* values did not clearly lie within the clusters. For the final SNP (753848-0_614), the within-genotype variation was smaller and clusters were quite distinct, with few uncalled genotypes, but the mean values of *p* were very different from 0.0, 0.5, and 1.0. The Welch statistics (τ_A*j*_, τ_B*j*_) for these four SNPs were (224.24, 155.44), (132.96, 46.95), (48.17, 35.44) and (58.97, 67.92), respectively. For the second SNP, there was a large difference between τ_A_, and τ_B_, with a clearer distinction between the means of the AA and AG genotypes than between the AG and GG genotypes.

For three of the 61 SNPs, the number of genotype calls was not sufficient to estimate the SNP parameters, and, for the remaining 58 SNPs, quantitative genotype probability matrices (quantitative genotypes) could be estimated only for 43% of individuals. The estimated error rates $\left(\widehat{\varepsilon}\right)$ of 47 SNPs were lower than 0.01, and the assumed error rates $\left(\tilde{\varepsilon}\right)$ for these were set to 0.01. For the remaining SNPs, seven had estimated error rates lower than 0.02, three had estimated error rates lower than 0.04, and one had an estimated error rate of 0.08.

For 87% of individuals, quantitative genotypes could be estimated on 50 or more SNPs, but for 10% of individuals, less than 20 SNPs provided quantitative genotypes. When quantitative genotypes could be estimated, in 95.4% of cases the most likely unordered genotype class had a genotype probability that exceeded 0.98, our threshold for declaring a genotype for use in the perturbed maximum likelihood and exclusion methods.

**Figure 2:**
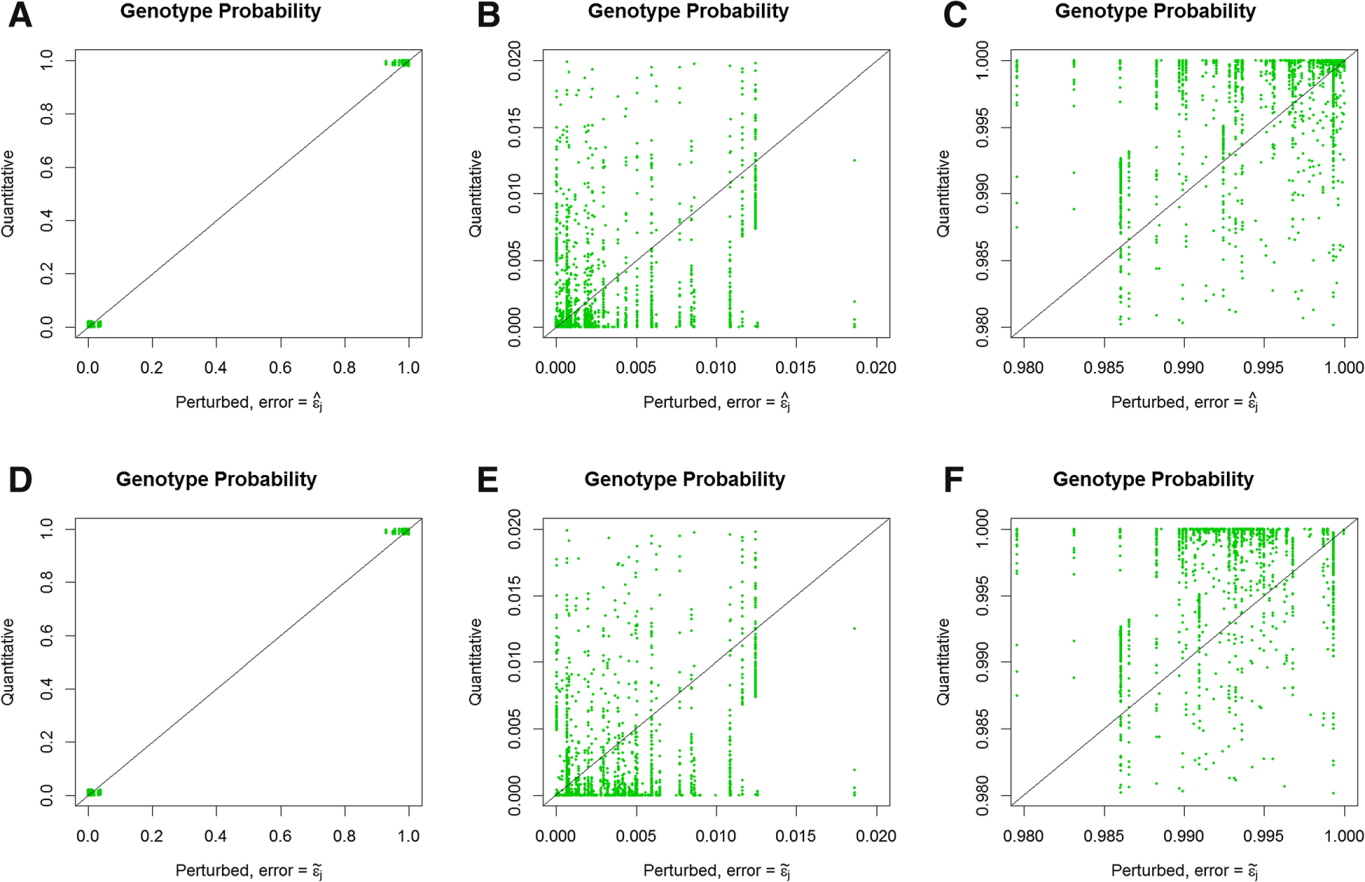
**Comparison of genotype probabilities for quantitative and perturbed genotypes.** Perturbed genotype probabilities were estimated using either an estimated error (top three panels) or an assumed error (bottom three panels), and were only estimated when the quantitative genotype probability exceeded 0.98; the data in the left hand panels **(A and** **D)** are expanded in the centre **(B and** **E)** and right hand panels **(C and** **F)**.

In Figure 2, quantitative genotype probabilities are compared to perturbed genotype probabilities. Generally, the quantitative genotype probability was far more certain (i.e., closer to 0 or 1) than the perturbed genotype probability. The patterns for estimated error and assumed error were remarkably similar; the effect of imposing a floor of 1% in the assumed error was reduced by multiplication by the expected genotype frequency given the allele frequencies. Consequently, although the assumed error rate was greater than or equal to 0.01, genotype probabilities were still commonly less than 0.01 or greater than 0.99. The patterns in panels A and D were affected by the threshold of 98% quantitative genotype certainty before a perturbed genotype was assigned, but a perturbed genotype was missing for that reason in only 3414 out of 72 039 cases.

### Parentage assignment

**Figure 3:**
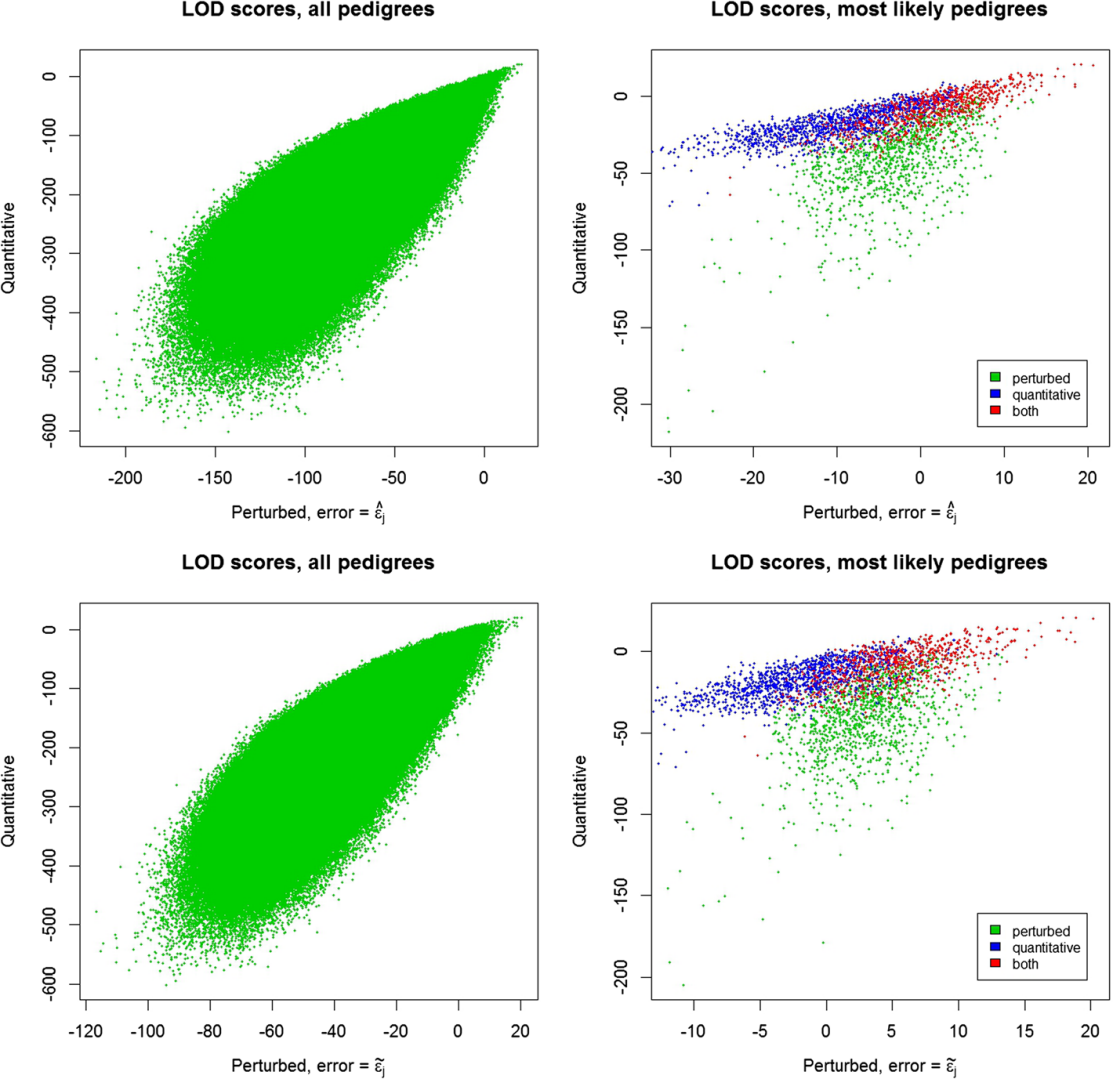
**Comparison of LOD scores obtained with quantitative and perturbed genotypes.** In the top two panels, the genotypes were perturbed (x-axis) using the estimated error rate, and in the bottom two panels, the genotypes were perturbed (x-axis) using the assumed error rate; the left hand panels contain LOD scores for all pedigrees, while in the right hand panels only LOD scores for the 10 most likely pedigrees for each progeny are plotted; these are coloured according to whether the pedigree appeared in the top 10 pedigrees obtained using perturbed genotypes, the top 10 pedigrees obtained using quantitative genotypes, or the top 10 pedigrees for both approaches.

G9 males (166) and females (124) recorded as potential parents of the G10 offspring were tested as parents of G10 progeny (210). Using the quantitative genotypes, of the 34 860 sire-offspring pairs, a LOD score was produced for only 28 566 pairs, the others having less than 10 SNPs for which both sire and offspring had an estimated allelic proportion. For dam-offspring pairs, 19 872 LOD scores were produced from a possible 26 040 pairs. Testing each sire by dam combination for the 207 offspring resulted in LOD scores for 2 741 922 sire-dam-offspring trios for which at least 10 SNPs in all three animals had a valid genotype. In Figure 3, LOD scores from quantitative and perturbed maximum likelihood are compared using the estimated error $\left(\widehat{\varepsilon}\right)$ and using the assumed error $\left(\tilde{\varepsilon}\right)$ for the perturbed maximum likelihood. The LOD scores that were estimated using the quantitative approach have the greatest range, followed by those estimated using the perturbed approach with an estimated error, followed by those estimated using the perturbed approach with an assumed error.

Differences in the distributions of LOD scores produced differences in the distribution of most likely pedigrees. Table 1 summarises, for each progeny, whether both parents appear to have genotype records included in the analysis, whether one parent appears to be missing, or whether both parents appear to be missing. Results differed largely between methods; exclusion allowing zero mismatches produced the least assignments and exclusion allowing three mismatches produced the most assignments. The three maximum likelihood methods were intermediate, with the quantitative method most similar to exclusion allowing zero mismatches, and the perturbed method with an assumed error most similar to exclusion allowing three mismatches. To understand the reasons for these differences, we examined individual cases when the perturbed method produced a high likelihood for a trio and the quantitative method did not. The differences were due to a small number of SNPs, which when perturbed moved genotype probabilities away from 0 or 1, while the quantitative estimates were very close to 0 or 1. In one example (progeny 2443, sire 1819, dam 1728), for SNP 78443-0_3537 (the first SNP in Figure 1, with error rates $\widehat{\varepsilon}=0.001,\kern0.37em \tilde{\varepsilon}=0.01$), the probabilities of the progeny genotype not being AA were < 1.0e-28, 0.0009 and 0.0091 for the quantitative and perturbation ($\widehat{\varepsilon}=0.001\ \mathrm{and}\ \tilde{\varepsilon}=0.01$) approaches respectively, and the probabilities of the sire genotype not being GG were <1.0e-14, 0.0005 and 0.0050. The effect of this SNP on the log likelihood was −31.9 for the quantitative genotype, −5.1 for the perturbed genotype ($\widehat{\varepsilon}=0.001$) and −2.9 for the perturbed genotype ($\tilde{\varepsilon}=0.01$) approaches. The penalty for an inconsistent genotype can be much larger for the quantitative approach, since the genotype probabilities can be much closer to 0 or 1.

**Table 1:** **Estimated counts of G10 offspring for which both sire and dam, only sire or only dam, or neither parent appeared to be present, depending on the method for parentage assignment used**

	**Method**				
**Most likely pedigree**	**Quantitative**	**Perturbed, error =** $\widehat{\boldsymbol{\varepsilon}}$	**Perturbed, error =** $\tilde{\boldsymbol{\varepsilon}}$	**Exclusion, zero mismatches**	**Exclusion, ≤ 3 mismatches**
Sire+dam	41	67	115	31	177
Sire+missing	151	133	91	66	0
Dam+missing	64	35	8	10	0
(sire or dam)+missing	56	31	7	81	30
Both missing	7	3	0	19	0

Counts are for the quantitative genotype method and perturbed genotype maximum likelihood method using either an estimated error ($\widehat{\boldsymbol{\varepsilon}}$) or an assumed error ($\tilde{\boldsymbol{\varepsilon}}$), and exclusion with a threshold of either zero or three mismatches. For sire+dam, the trio LOD is greater than 0, the sire LOD and the dam LOD. For sire+missing, the sire LOD is greater than both 0 and the trio LOD, and the dam LOD is less than the trio LOD or less than 0. For dam+missing, the dam LOD is greater than both 0 and the trio LOD, and the sire LOD is less than the trio LOD or less than 0. For (sire or dam)+missing, the sire LOD and dam LOD are both greater than 0, and both greater than the trio LOD. If all LOD scores are less than 0, it is assumed that genotypes for both parents are missing. Equivalent conditions are applied for the exclusion method.

**Figure 4:**
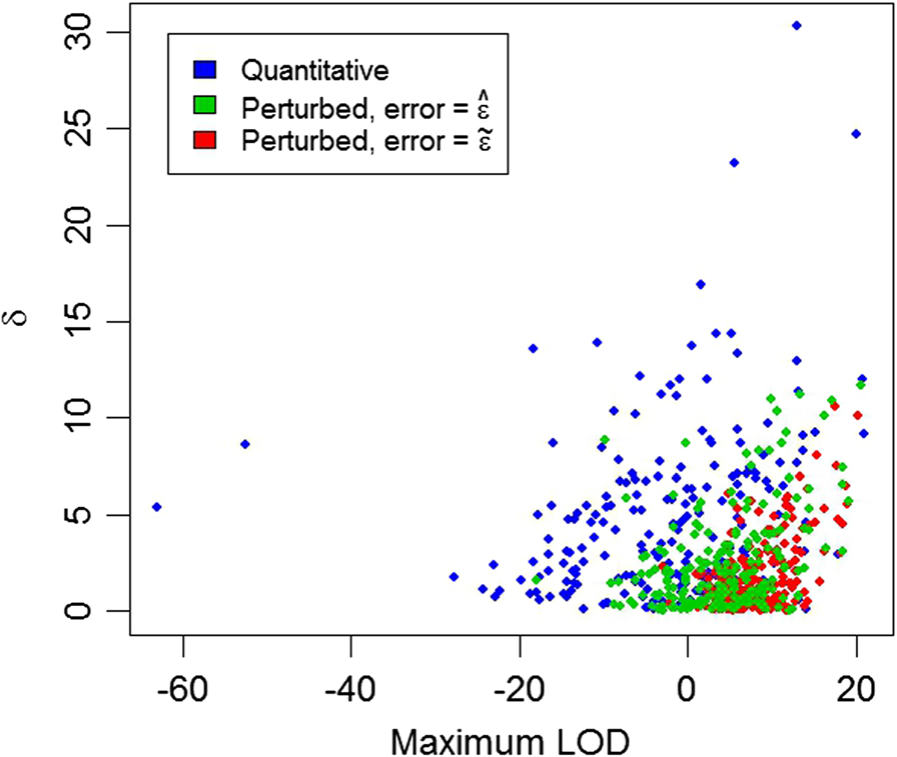
**Comparison of LOD scores for most likely pedigrees obtained with quantitative and perturbed genotypes.** For each progeny, the LOD score of the most likely sire-dam family (x-axis) and δ, the difference between the LOD for the most likely and second most likely sire dam family (y-axis), are plotted; colours indicate whether quantitative or perturbed (estimated error or assumed error) genotypes were used.

In Figure 4, for each progeny, the LOD scores for the most likely pedigree is compared to δ, the difference in LOD between the most likely and second most likely pedigrees. For arbitrary thresholds of maximum LOD greater than 3.0 and δ greater than 3.0, assignment rates for the quantitative maximum likelihood and perturbed maximum likelihood ($\widehat{\varepsilon}=0.001\ \mathrm{and}\ \tilde{\varepsilon}=0.01$) approaches were similar at 23, 22 and 20% respectively. However, the families assigned under the approaches frequently differed; for only 9% of progeny did the quantitative and perturbed approaches most exceed the thresholds for the same parent pair, regardless of whether the estimated or assumed error rate was used for the perturbed approach.

**Figure 5:**
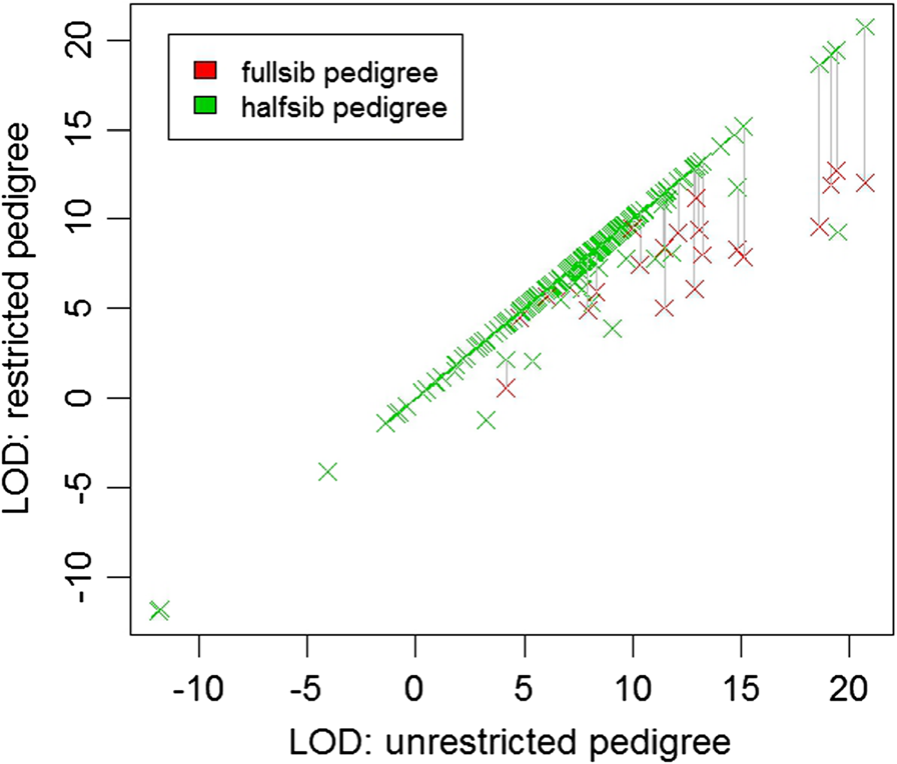
**Effect of constraining the pedigree.** The maximum sire-dam-offspring trio LOD score is plotted for each of the 207 G10 progeny; on the y-axis, the maximum LOD score for the unrestricted pedigree is plotted, while on the x-axis the maximum LOD scores for the half-sib and full-sib pedigrees are plotted; the order of plotting is full-sibs followed by half-sibs; full-sib data points are masked by half-sib data points, except when the two differ.

The remaining results relate to the quantitative genotypes and likelihoods. Summaries of the impact of the pedigree constraints are in Table 2. The three largest families were the same for all three analyses, each comprising a known sire and unknown dam. In Figure 5, the LOD scores for unrestricted pedigrees are compared to LOD scores for full-sib and half-sib pedigrees. Most of the increase in LOD score observed when going from full-sib families to half-sib families occurred due to a small number of individuals, each with a large LOD score, leaving a full-sib family to form their own half-sib family. The quality of the individual genotype, measured as the sum of the genotype probabilities for the most likely unordered genotype for each SNP, had no effect on whether individuals were assigned to the same or different parents in the half-sib and unrestricted pedigrees (P > 0.3), but there was a suggestion that it had an effect on whether individuals were assigned to the same or different parents in the full-sib and half-sib pedigrees (P = 0.08, residual deviance 133.17 on 205 degrees of freedom).

**Table 2:** **Estimates of numbers of families, sires and dams for the unrestricted, half sib and full sib pedigrees**

	**Families**	**Sires**	**Missing sires**	**Dams**	**Missing dams**	**Largest family**	**LOD**
Unrestricted	64	42	16	47	20	52	1,529
Half sib	51	44	18	44	20	53	1,481
Full sib	43	43	17	43	21	55	1,397

Counts for sires and dams include those that are missing, and the LOD is the sum of the LOD scores for the individual assignments.

### Estimation of allele frequencies for pooled samples

**Figure 6:**
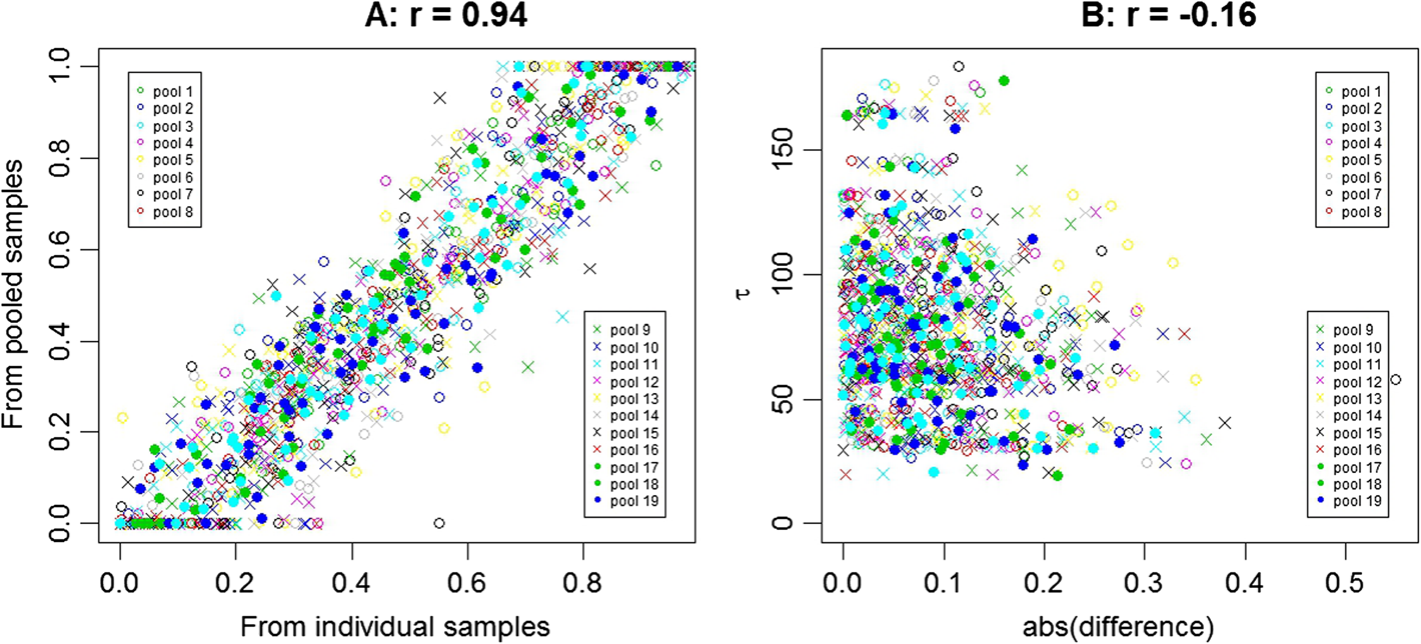
**Accuracy of allele frequencies estimated from pooled DNA.** SNP allele frequencies estimated from pooled or individual samples are compared in panel **A**, and, in panel **B**, the absolute value of the difference between allele frequency estimates from pooled and individual samples is plotted as a function of the estimate of the relevant Welch statistic (τ).

Genotyping of three of the 22 pools failed and only results for the other 19 pools are presented, in Table 3 and graphically in Figure 6. The proportion of genotypes available for individuals known to be in pools differed between pools, with a higher average proportion of individual shrimp genotypes available for pools with G10 individuals. The G9 and G10 samples were taken a year apart, and DNA was extracted soon after collection, so there was a batch effect of unknown cause. Not surprisingly, differences in the proportion of genotypes available for individuals known to be in the pools had a significant effect on the correlation between $\widehat{f}$ and *f* * (P = 0.034). Generation (G9 or G10) only affected the correlation through its effect on the proportion of genotypes available on individuals; if this proportion was in the model then the generation effect on the correlation was not significant. There was also variation in the number of SNP genotypes available per pool, and this also had an effect on the correlation (P = 0.002). This suggests that for assays with fewer SNPs called, results for other SNPs were also more variable. The estimate of the Welch statistic (Figure 6, panel B) had a highly significant effect on the correspondence between $\widehat{f}$ and *f* * (P < 1e-7); the difference between $\widehat{f}$ and *f* * tended to be smaller for estimates with high Welch statistics (i.e. those in intervals where genotype clusters for samples from individual DNA were clearly distinguished).

**Table 3:** **Correlation between allele frequencies estimated from pooled samples (** $\widehat{\boldsymbol{f}}$ **) and allele frequencies estimated from individual samples (** ***f*** *)

**Pool**	**Shrimp**	**Generation**	**Shrimp genotyped**	**SNP in** $\widehat{\boldsymbol{f}}$	$\boldsymbol{r}\left(\widehat{\boldsymbol{f}},{\boldsymbol{f}}^{*}\right)$
1	24	G10	98%	50	0.97
2	24	G10	98%	51	0.96
3	23	G10	97%	52	0.97
4	22	G10	95%	52	0.95
5	22	G10	99%	41	0.9
6	22	G10	98%	51	0.94
7	23	G10	93%	41	0.92
8	18	G10	98%	52	0.98
9	24	G9	65%	51	0.9
10	24	G9	73%	49	0.93
11	24	G9	66%	52	0.93
12	23	G9	70%	48	0.95
13	24	G9	68%	49	0.95
14	24	G9	78%	46	0.92
15	24	G9	83%	48	0.92
16	23	G9	75%	50	0.94
17	24	G9	86%	48	0.95
18	24	G9	65%	52	0.93
19	24	G9	79%	50	0.94

“Shrimp genotyped” is the mean number of shrimp contributing to the estimate of *f* *, expressed as a percentage of the number of shrimp in the pool, and “SNP in $\widehat{f}$” is the number of SNP without missing values in $\widehat{f}$.

### Estimation of family contributions to pools

**Figure 7:**
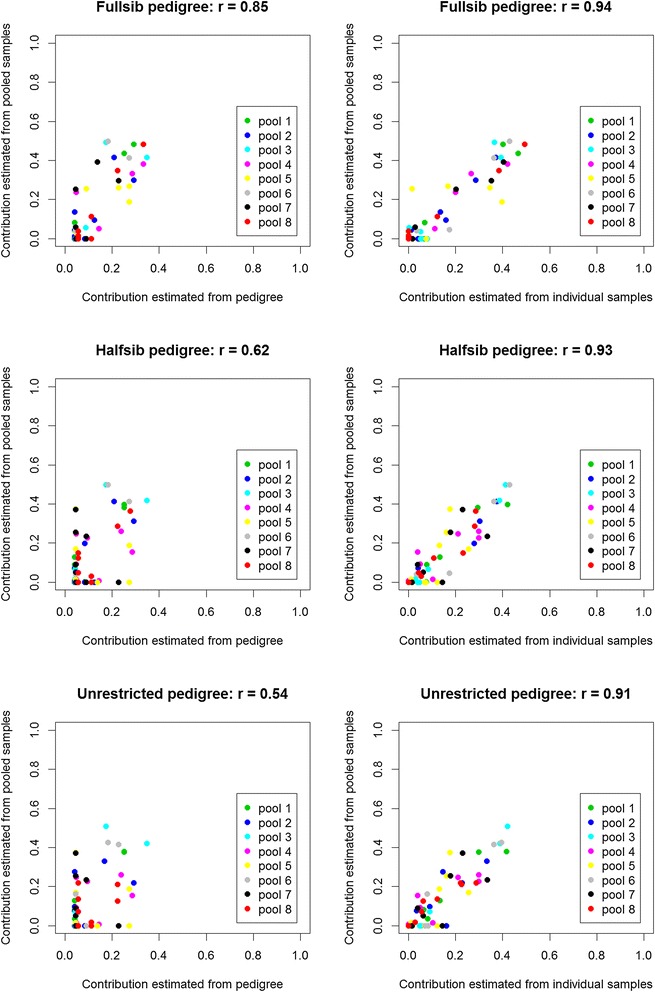
**Estimation of family contributions to pools.** Estimates of family contributions to pools from pooled DNA samples (y-axis) are compared to family contributions to pools estimated from pedigree (x-axis, left hand panels) and to family contributions estimated from individual DNA samples (x-axis, right hand panels).

For the eight G10 pools, family contributions estimated from pedigree ranged from 4 to 35% (1/24 to 8/23). The most abundant family generally accounted for between one quarter and one third of the individuals in a pool (Table 4). The correlation between the contributions estimated from pedigree and from the pooled DNA samples (i.e. using $\widehat{f}$) differed based on the pedigree used (Figure 7, left hand panels), with the highest correlation (0.85) for the full-sib pedigree and the lowest correlation (0.54) for the unrestricted pedigree. Contributions of the larger families were over-estimated with the pooled DNA samples and contributions of the smaller families were under-estimated. If family contributions were estimated from the mean value of *p* for individuals in the pool (i.e. using  *f* *) instead of from pooled DNA samples, correlations with pedigree-based estimates were 0.87, 0.65 and 0.60 for the full-sib, half-sib and unrestricted pedigrees, respectively. These correlations did not differ greatly from the correlations obtained from pooled DNA samples, which may be explained by the high correlation between contributions estimated from pooled and from individual samples (Figure 7, right hand panels).

**Table 4:** **Estimated family contributions to pools based on the full sib, half sib and unrestricted pedigrees**

		**Full sib**		**Half sib**		**Unrestricted**	
**Pool**	**Shrimp**	**Families**	**Max (contrib)**	**Families**	**Max (contrib)**	**Families**	**Max (contrib)**
1	24	12	0.29	13	0.25	14	0.25
2	24	11	0.29	10	0.29	13	0.29
3	23	12	0.35	13	0.35	13	0.35
4	22	8	0.33	9	0.29	9	0.29
5	22	7	0.27	9	0.27	9	0.27
6	22	11	0.27	12	0.27	14	0.23
7	23	14	0.23	16	0.23	17	0.23
8	18	8	0.33	9	0.28	10	0.22

## Discussion

This study focussed on the inference of relationships between individuals using genotype data obtained from a low-cost low-density genotyping platform. We used an example from aquaculture but the results are also relevant to other industries for which the value of an individual animal is low. We chose to restrict our genotype data to that obtainable from a single low-density Sequenom 63-SNP multiplexed assay, the cost of which is similar to that of genotyping a very small number of multiplexed microsatellites (e.g. two panels of six and seven microsatellites, as used by Sellars *et al.* [32]). Rather than increase power and cost by using multiple Sequenom assays to genotype additional SNPs, we used statistical approaches to extract more information from the data available from an existing single SNP assay. Notably, quantitative genotype estimates were used in data processing, rather than genotypes that had been classified into discrete genotype classes. This also meant that extension to analysis of DNA from groups of individuals was straightforward.

The differences in the pattern of area measurements between SNPs (Figure 1) provide immediate justification for treating SNP genotypes as quantitative values. With the assay used in this study, it is clear that there is often considerable variability amongst X-Y signals within a genotype cluster, while other clusters form very tight groups. We chose to use a normal distribution to model the distribution of area measurements, for consistency with algorithms used to call alleles, but another distribution is clearly more appropriate for some alleles and some SNPs. We recognise that the benefits of quantitative genotypes may be less for other, more accurate genotyping platforms, or for Sequenom panels with more rigorous SNP screening. The panel used here, although of modest power, was very affordable in terms of both development and application. However, we do believe that investment in better SNP screening is often justified. As with the use of statistical approaches to increase power, and in contrast to using multiple panels to increase power, once development costs have been accounted for, an up-front investment in SNP screening produces no ongoing additional cost per sample.

We tried to keep our approach to parentage assignment as clear as possible. The Mendelian transmission rules are simple and constant for diploid species, and are easily represented by matrix operations, regardless of whether genotypes are discrete or continuous. The error function we used for our quantitative approach assumed that area estimates produced by the genotyping platform have a normal distribution. Alternative maximum likelihood approaches make different assumptions about the distribution of the error, but the matrix algebra is the same. One characteristic of our error distribution is that, unlike (for example) Marshall *et al.* [19], we did not allow for additional errors associated with the possibility of sample swaps. Because the Sequenom assay is applied to all SNPs in parallel, all SNP genotypes come from the same DNA sample. Thus, a sample swap will either be identified due to inconsistencies with the known pedigree, or if it goes unnoticed, its impact will be equivalent to a tagging error.

We observed large differences between results for the quantitative approach and the perturbed error approach in our study, which highlights the importance of the error term in the maximum likelihood procedure. For some SNPs and some individuals, the quantitative approach sets the error term to a very low value, essentially zero, and therefore applies a strict exclusion criterion for these SNPs. For other SNPs and individuals, a more lenient error term is applied. Whether this is better or worse than SNP specific error rates, or the arbitrary error rate of 1% used for comparison with perturbation, depends on how well the alternatives match the true but unknown error distribution. For a SNP genotyping technology for which the probability of the relevant homozygous genotype is effectively equal to 0 whenever the signal for either X or Y is effectively 0, the quantitative method is preferred. If there is a probability that at random one of the X or Y signals totally drops out for a heterozygous individual, while the other signal remains strong, then the perturbed method is preferred. There is nothing in our dataset to help us to choose between these two alternatives. The assignment rates for the two approaches were both unacceptably low at around 20%, due either to a lack of power in the panel or missing genotypes on parents. However, because the families assigned using one error distribution had only a small overlap with the families assigned using other error distributions, it is clearly very important that we learn all we can about the true distribution of errors.

Based on Figure 5, it is apparent that, the application of constraints to the pedigree in the second stage of parent assignment to account for biological constraints and hatchery records affected only a relatively small number of individuals. In the unconstrained pedigree, most offspring were assigned to full-sib families, which is consistent with our understanding of the data. For the constrained pedigrees, without access to a more powerful DNA parentage panel, it is not possible to determine whether departures from a full-sib model are due to parents having multiple mates, or to incorrect assignments. The weak association between the quality of the individual’s genotype data and consistency of assignment between full-sib and half-sib families suggests that the departures from a full-sib model may be at least partly due to incorrect assignments caused by poor quality genotype data.

A weakness of this study is the lack of knowledge on the true parentage of the G10 progeny, especially for families for which a parent was declared as missing. We do not know whether the true parent was not tested, or whether the true parent was tested but genotyping failed (which occurred in around 10% of cases), or whether the true parent was tested and wrongly excluded. However, for industrial application in the *P. monodon* breeding program, although not desirable, this incomplete information is not an insurmountable problem. Individuals in such families, while missing one parent, are known to be full-sibs, and the potential mates of the identified parent are likely known and from a common genetic background. Thus, it is unlikely that breeding decisions would differ in a significant way if the missing information was available.

Correlations between allele frequencies estimated from individual and from pooled samples were consistently greater than 0.9. Not surprisingly, given the variability between SNPs (Figure 1), there was a strong association between the Welch statistics (τ_A*j*_ or τ_B*j*_) related to the interval that contained the allele frequency estimate (*p*_*kj*_) from pooled DNA, and the absolute value of the difference between the allele frequency estimate from pooled DNA and from individual DNA (Figure 6, panel B). This suggests that for low-density SNP assays, variation in SNP quality is as important as other forms of variation when analysing samples from pooled DNA.

The high correlations between allele frequency estimates from pooled and individual samples were reflected in the high correlations between estimates of family contributions to pools when using allele frequencies from the two sources (Figure 7, right hand panels). The correlations between family contributions to pools estimated from pedigree and from allele frequencies were not as high (Figure 7, left hand panels). This indicates that 50 SNPs is too few to produce accurate estimates of contributions for pools containing individuals from around 12 families. However, especially in the case of the full-sib pedigree, the correlations were positive, and probably high enough to provide useful information. Despite this, because much fewer assays are needed when DNA samples are pooled, it will be cost effective to use multiple low-density SNP assays such as the four *P. monodon* multiplexed assays already available [39] or high-density SNP assays when they become available.

## Conclusions

Treating SNP genotypes as continuous instead of as discrete values in parentage assignment poses no additional statistical problems. The maximum likelihood framework seamlessly incorporates quantitative allele probabilities; they only require an alternative formulation for the distribution of the error term. However, since the distribution of the error term can have a large impact on pedigree assignments, it is important to gain an understanding of this distribution. The likelihood equations can be conveniently formulated using matrix algebra, allowing easy implementation on any software platform that supports matrix operations. Using the Sequenom assay with only 63 SNPs, most individuals were assigned to full-sib families, as expected given the origin of the samples. Further refinement of the pedigree by constraining parents to only one mate reduced the number of families from 64 to 43, and probably results in a more accurate pedigree given the uncertainty in the parentage assignments from a low-density SNP panel. Estimates of family contributions in pooled samples of DNA obtained with 63 SNPs were of low accuracy, and for this application the use of multiple multiplexed low-density SNP assays would be beneficial.

## Appendix

**Worked example**

***Estimation of SNP specific parameters***

Additional file 1: Figure S1 illustrates the mapping from Cartesian coordinates to polar coordinates for SNP 186827-0_535. In this example, the X and Y areas that relate to SNP alleles A and G, are adjusted for the area uncertainties. The plots highlight the data points for the sire, dam and offspring used throughout this Appendix to illustrate the method. Adjusted X and Y values are (36.6, 13.4), (25.3, 22.9) and (30.4, 0) for the sire, dam and offspring, respectively.

SNP specific parameters for SNP 186827-0_535 are in Additional file 2: Table S1. For the sire, the estimate of allelic proportion is

$$ \begin{array}{c}\hfill {p}_{sj}=\frac{{ \tan}^{-1}\left(\frac{13.4}{36.6}\right)}{\frac{\pi }{2}}\hfill \\ {}\hfill =0.223.\hfill \end{array} $$

Similarly, values for the dam and offspring are equal to 0.468 and 0.000, respectively.

***Calculation of parentage likelihood***

For this SNP, the alleles are A and G, and for the sire:

$$ \begin{array}{cc}\hfill {\Phi}_{\mathrm{AA}} = {\varphi}_{0.036,0.057}(0.223)\hfill & \hfill = 0.033\hfill \\ {}\hfill {\Phi}_{\mathrm{AG}} = {\Phi}_{\mathrm{GA}}=\frac{\varphi_{0.400,0.137}(0.223)}{2}\hfill & \hfill = 0.633\hfill \\ {}\hfill {\Phi}_{\mathrm{GG}} = {\varphi}_{0.742,0.096}(0.223)\hfill & \hfill =0.000\hfill \end{array}, $$

where *φ*_*μ*,*σ*_(*x*) is the height of the normal (*μ*, *σ*) distribution at *x*. Scaling to make the sum equal to 0 gives the genotype probability matrix for the sire:

$$ {\mathbf{G}}^{\mathbf{sj}}=\frac{\left[\begin{array}{cc}\hfill {\Phi}_{\mathrm{AA}}\hfill & \hfill {\Phi}_{\mathrm{AG}}\hfill \\ {}\hfill {\Phi}_{\mathrm{GA}}\hfill & \hfill {\Phi}_{\mathrm{GG}}\hfill \end{array}\right]}{{\displaystyle \sum }{\Phi}_{ij}} = \frac{\left[\begin{array}{cc}\hfill 0.033\hfill & \hfill 0.633\hfill \\ {}\hfill 0.633\hfill & \hfill 0.000\hfill \end{array}\right]}{1.299}=\left[\begin{array}{cc}\hfill 0.025\hfill & \hfill 0.487\hfill \\ {}\hfill 0.487\hfill & \hfill 0.000\hfill \end{array}\right]. $$

It is most likely that the sire is heterozygous AG but there is a small probability that it is homozygous AA. Likewise, **G**^**dj**^ and **G**^**oj**^ are

$$ \left[\begin{array}{cc}\hfill 0.000\hfill & \hfill 0.487\hfill \\ {}\hfill 0.487\hfill & \hfill 0.026\hfill \end{array}\right]\ \mathrm{and}\ \left[\begin{array}{cc}\hfill 0.993\hfill & \hfill 0.004\hfill \\ {}\hfill 0.004\hfill & \hfill 0.000\hfill \end{array}\right], $$

respectively.

The transmission vector for the sire is:

$$ {\mathbf{T}}^{\mathbf{s}\mathbf{j}}=\frac{\left({\mathbf{G}}^{\mathbf{s}\mathbf{j}}\left[\begin{array}{c}\hfill 1\hfill \\ {}\hfill 1\hfill \end{array}\right]+{\mathbf{G}}^{\mathbf{s}{\mathbf{j}}^{\hbox{'}}}\ \left[\begin{array}{c}\hfill 1\hfill \\ {}\hfill 1\hfill \end{array}\right]\right)}{2} = \left[\begin{array}{c}\hfill 0.513\hfill \\ {}\hfill 0.487\hfill \end{array}\right], $$

and the transmission vector for the dam is $$ {\mathbf{T}}^{\mathbf{dj}}=\left[\begin{array}{c}\hfill 0.487\hfill \\ {}\hfill 0.513\hfill \end{array}\right] $$ so the transmission matrix for the sire dam pairing is:

$$ {\mathbf{T}}^{\mathbf{sj}}{\mathbf{T}}^{\mathbf{d}{\mathbf{j}}^{\hbox{'}}}=\left[\begin{array}{cc}\hfill 0.250\hfill & \hfill 0.263\hfill \\ {}\hfill 0.237\hfill & \hfill 0.250\hfill \end{array}\right]. $$

The likelihood of the trio (*s, d, o*) for marker *j* is:

$$ \begin{array}{c}\hfill {L}^{(sdo)j}=\mathrm{sum}\left(\left({\mathbf{T}}^{\mathbf{sj}}{\mathbf{T}}^{\mathbf{d}{\mathbf{j}}^{\hbox{'}}}\right){}^{\circ}{\mathbf{G}}^{\mathbf{oj}}\right)\hfill \\ {}\hfill =\mathrm{sum}\left(\left[\begin{array}{cc}\hfill 0.250\hfill & \hfill 0.263\hfill \\ {}\hfill 0.237\hfill & \hfill 0.250\hfill \end{array}\right]\circ \left[\begin{array}{cc}\hfill 0.993\hfill & \hfill 0.004\hfill \\ {}\hfill 0.004\hfill & \hfill 0.000\hfill \end{array}\right]\right)\hfill \\ {}\hfill =\mathrm{sum}\left[\begin{array}{cc}\hfill 0.248\hfill & \hfill 0.001\hfill \\ {}\hfill 0.001\hfill & \hfill 0.000\hfill \end{array}\right]\hfill \\ {}\hfill =0.250\hfill \end{array}. $$

The likelihood *L*^*oj*^ is the likelihood under the null hypothesis that the offspring is unrelated to the sire and dam, and is equal to the likelihood for a random parental pair given the allele frequencies. For this SNP, the frequency of the G allele is equal to 0.354, so the transmission vector for a random parent is:

$$ {\mathbf{F}}^{\mathbf{j}}=\left[\begin{array}{c}\hfill 0.646\hfill \\ {}\hfill 0.354\hfill \end{array}\right], $$

and the transmission matrix for a random sire dam pairing is:

$$ {\mathbf{F}}^{\mathbf{j}}{\mathbf{F}}^{{\mathbf{j}}^{\hbox{'}}}=\left[\begin{array}{cc}\hfill 0.418\hfill & \hfill 0.229\hfill \\ {}\hfill 0.229\hfill & \hfill 0.125\hfill \end{array}\right]. $$

The likelihood that the offspring comes from a random pair of parents is:

$$ {L}^{oj}=\mathrm{sum}\left(\left({\mathbf{F}}^{\mathbf{j}}{\mathbf{F}}^{{\mathbf{j}}^{\hbox{'}}}\right)\circ {\mathbf{G}}^{\mathbf{oj}}\right)=0.416. $$

The log of the likelihood ratio is $\log \left(\frac{L^{(sdo)j}}{L^{oj}}\right) = -0.511$. The likelihood for a random pair of parents is greater than the likelihood of the sire dam pair for this offspring for this SNP, so the log of the likelihood ratio is less than 0.

***Estimation of allele frequencies for pooled samples***

At this same SNP (*j*), for a pool (*t*) with allelic proportion *p*_*tj*_ = 0.289, because *μ*_AA*j*_ < *p*_*tj*_ < *μ*_AB*j*_,

$$ \begin{array}{l}\widehat{f_{tJ}}=0.5\left(\frac{\left({p}_{tj}-{\mu}_{\mathrm{AA}j}\right)}{\left({\mu}_{\mathrm{AB}j}-{\mu}_{\mathrm{AA}j}\right)}\right)\hfill \\ {}=0.5\left(\frac{\left(0.289-0.036\right)}{\left(0.400-0.036\right)}\right)\hfill \\ {}=0.348\hfill \end{array} $$

and the Welch statistic associated with $\widehat{f_{tJ}}$ is equal to 48.170.

## Additional files

Additional file 1: Figure S1 Example of conversion from Cartesian to polar coordinates. Data points for SNP 186827-0_535 are plotted in Cartesian coordinates and in polar coordinates. Intensities in the polar coordinate plot are estimated as Euclidean distances from the origin to the data points in Cartesian coordinates; identified in the plot are the sire, dam and offspring used to demonstrate the method. Additional file 2: Table S1 Contains the numbers of data points (n), the mean values of allelic proportion *p* (μ) and standard deviations of *p* (σ), for genotype classes AA, AG and GG. The Welch statistics (τ) for testing means of *p* are also included for genotypes AA and AG, and genotypes AG and GG.
