# Supplementary figures and images for: Quantitative analysis of low-density SNP data for parentage assignment and estimation of family contributions to pooled samples
Source: Genet Sel Evol. 2014 Sep 2;46(1):51. doi: 10.1186/s12711-014-0051-y (PMC4244062; doi:10.1186/s12711-014-0051-y)

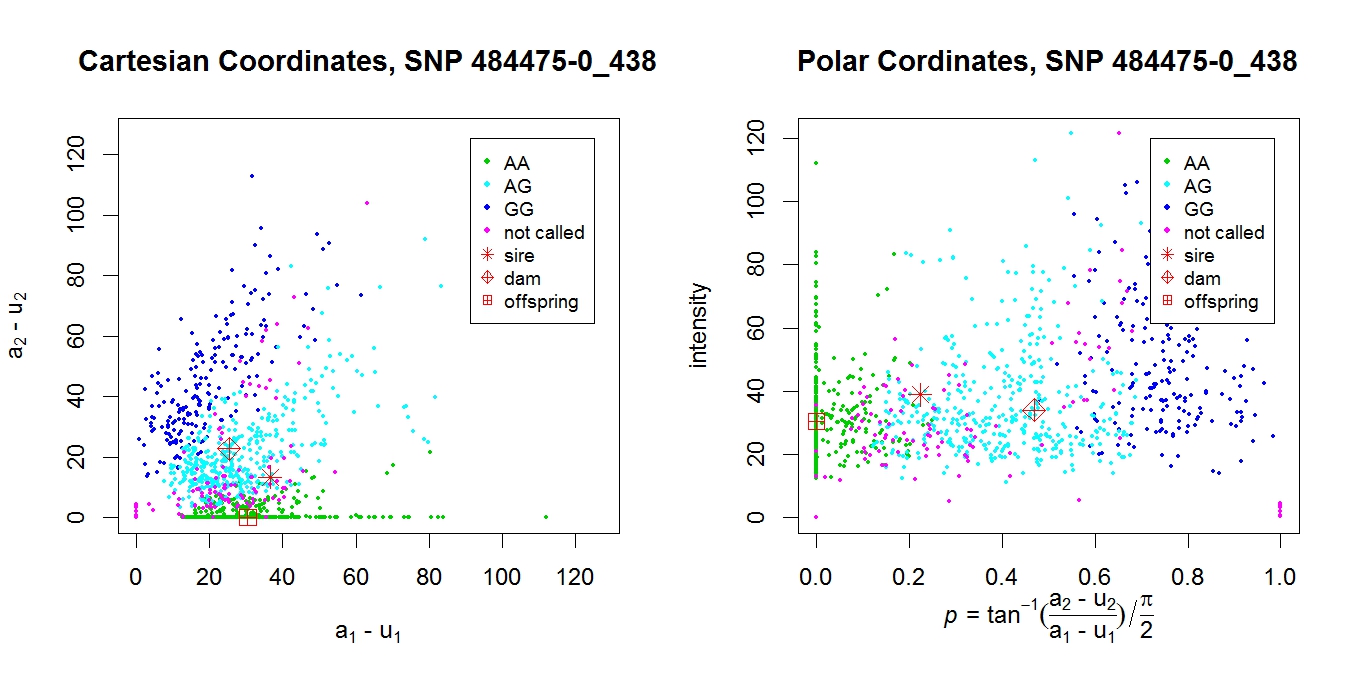

Supplement: Additional file 1: Figure S1 — Example of conversion from Cartesian to polar coordinates. Data points for SNP 186827-0_535 are plotted in Cartesian coordinates and in polar coordinates. Intensities in the polar coordinate plot are estimated as Euclidean distances from the origin to the data points in Cartesian coordinates; identified in the plot are the sire, dam and offspring used to demonstrate the method. [file 12711_2014_51_MOESM1_ESM.jpeg]
