# Supplementary material for: Quantitative analysis of low-density SNP data for parentage assignment and estimation of family contributions to pooled samples
Source: Genet Sel Evol. 2014 Sep 2;46(1):51. doi: 10.1186/s12711-014-0051-y (PMC4244062; doi:10.1186/s12711-014-0051-y)
Supplement: Additional file 2: Table S1 — Contains the numbers of data points (n), the mean values of allelic proportion p (μ) and standard deviations of p (σ), for genotype classes AA, AG and GG. The Welch statistics (τ) for testing means of p are also included for genotypes AA and AG, and genotypes AG and GG. [file 12711_2014_51_MOESM2_ESM.docx]

| **Genotype** | **n** | $\boldsymbol{\mu}$ | $\boldsymbol{\sigma}$ | $\boldsymbol{\tau}$ |
| --- | --- | --- | --- | --- |
| AA | 297 | 0.036 | 0.057 |  |
| AA to AG |  |  |  | 48.170 |
| AG | 407 | 0.400 | 0.137 |  |
| AG to GG |  |  |  | 35.444 |
| GG | 195 | 0.742 | 0.096 |  |

Additional file 2: Table S1
